# What’s New with TAVR? An Update on Device Technology

**DOI:** 10.14797/mdcvj.1230

**Published:** 2023-05-16

**Authors:** Syed Zaid, Marvin D. Atkins, Neal S. Kleiman, Michael J. Reardon, Gilbert H. L. Tang

**Affiliations:** 1 Houston Methodist DeBakey Heart & Vascular Center, Houston Methodist, Houston, Texas, US; 2 Mount Sinai Medical Center, New York, US

**Keywords:** aortic stenosis, transcatheter aortic valve implantation, prosthetic aortic valves, aortic valve replacement

## Abstract

Over the last 20 years, transcatheter aortic valve replacement (TAVR) has revolutionized the management of aortic stenosis and has become the standard of care across the entire spectrum of surgical risk. Expansion of TAVR in treating younger, lower-risk patients with longer life expectancies, and treating earlier in the disease process, has seen a continuous evolution in device technology, with several next-generation transcatheter heart valves developed to minimize procedural complications and improve patient outcomes. This review provides an update on the latest advances in transcatheter delivery systems, devices, and leaflet technology.

## Introduction

Transcatheter aortic valve replacement (TAVR) was originally designed for patients with severe aortic stenosis (AS) who were considered inoperable.^1^ Since the first in-human TAVR performed in 2002 via a transseptal approach in a patient with severe AS and cardiogenic shock, TAVR has expanded today to younger and lower-risk patients, regardless of surgical risk.^2^,^3^ Evolution of TAVR technology has also expanded its reach to newer patient populations with a wider range of anatomies. According to a 2020 report from the Society of Thoracic Surgeons-American College of Cardiology Transcatheter Valve Therapy (STS-ACC TVT) Registry, TAVR volumes increased every year from 2011 to 2019, exceeding surgical aortic valve replacement in 2019.^4^ The number of sites performing TAVR also has increased, with the procedure now performed in all 50 states.

As TAVR technology has evolved, innovations in transcatheter heart valve (THV) design and delivery systems have reduced procedural complications and improved clinical outcomes.^5^,^6^,^7^ Since 2011, TAVR has seen a reduction in 30-day mortality (7.2% to 2.5%), and stroke (2.75% to 2.3%), with acceptable patient-reported outcomes achieved in 8 of 10 patients at 1 year.^4^ While improvements in THV sizing and design have reduced paravalvular leak (PVL) and conduction disturbances, lower profile delivery systems with greater procedural capability through the transfemoral route have mitigated vascular complications and bleeding.^8^ Balloon-expandable valves (BEV) and self-expanding valves (SEV) have emerged as the two dominant THV platforms, and THV features have been iterated over each generation for both platforms. In this article, we provide an update on the key advancements in the new generation THVs and delivery systems for the various BEV and SEV platforms available commercially or in trials in the US.

## Transcatheter Heart Valves

### SAPIEN X4 with Resilia Valve

Over the past 20 years, the evolution of Edwards’ balloon-expandable TAVR technology has been remarkable, with the development of five generations of SAPIEN prostheses, the SAPIEN X4 being the latest iteration. The intra-annular balloon-expandable Edwards THV has evolved from a stainless-steel tubular frame with equine pericardial tissue leaflet to a more sophisticated cobalt-chromium frame with an outer skirt and a bovine pericardial tissue leaflet. Likewise, the Edwards delivery system also has evolved from a simple valvuloplasty balloon to the latest-generation delivery system that comprises mechanisms for aligning the THV on the balloon, thus facilitating delivery through smaller sheaths (up to 14F). Compared with the previous-generation S3, the fourth-generation SAPIEN 3 Ultra (SAPIEN 3U), while maintaining the same stent frame design, had a higher sealing skirt made of textured polyethylene terephthalate extending approximately 40% higher above the valve inflow, which has further reduced the incidence of PVL.^9^,^10^ The latest iteration of the Edwards THV, the fifth generation SAPIEN X4 THV, has been redesigned with a focus on lifetime management of aortic stenosis.

**Figure 1:**
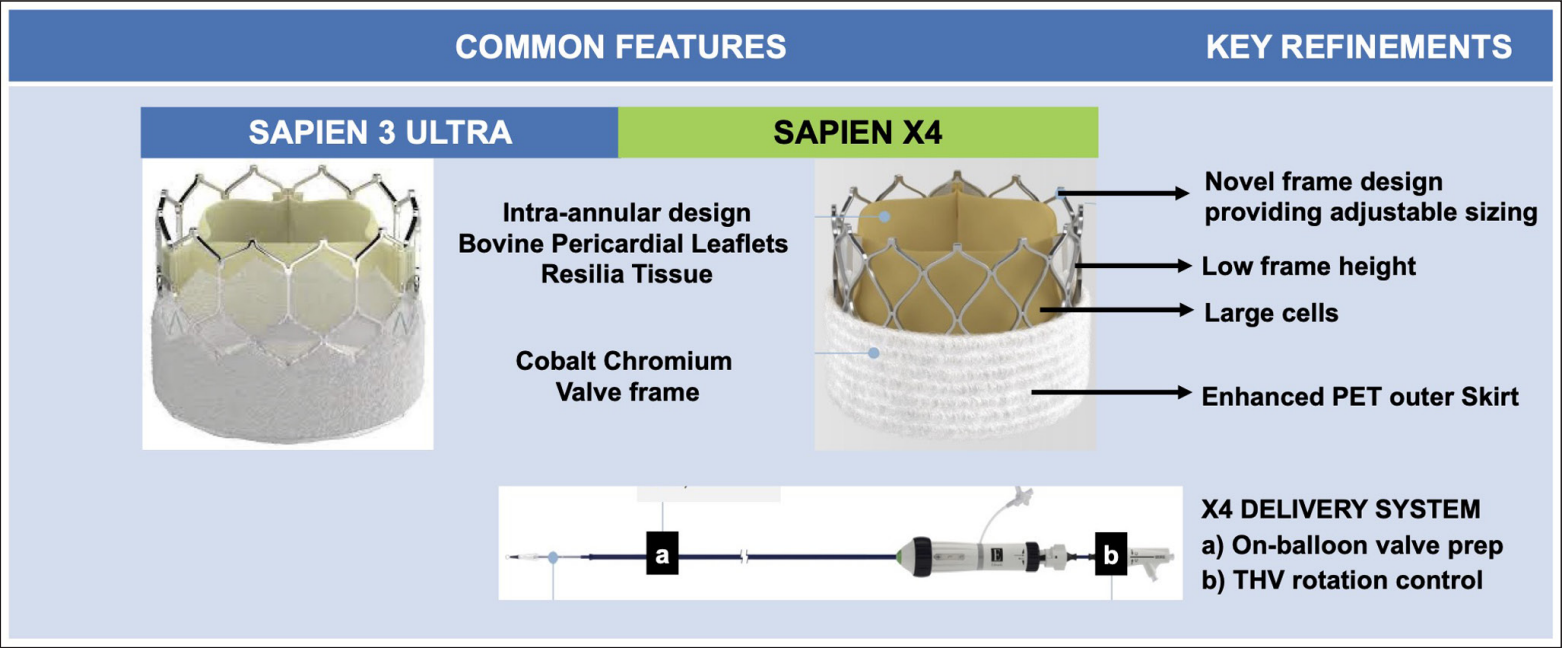
Novel features in the SAPIEN 3 Ultra versus SAPIEN X4 transcatheter heart valves.

The SAPIEN X4 system has the following novel advancements compared with the SAPIEN 3U system (Figure 1):

1. Enhanced anticalcification technology with the RESILIA tissue platform to extend THV longevity, which also enables dry storage and maintains bovine pericardial leaflets matched for thickness and elasticity.
2. Novel frame design that enables adjustable sizing while maintaining high radial strength and valve performance over the deployment diameter range. The three available THV sizes include 23 mm, 26 mm, and 29 mm, with 16 unique deployment diameters in 0.5-mm increments, supporting treatment of aortic valves with an annular diameter of 21 mm to 24 mm, 24 mm to 27 mm, and 27 mm to 30 mm, respectively.
3. Shorter frame height and large cells to facilitate future coronary access.
4. Enhanced polyethylene terephthalate (PET) outer skirt designed to further minimize PVL while maintaining low profile access.
5. SAPIEN X4 delivery system with THV rotation control and on-balloon valve preparation that enables independent control of valve orientation and commissural alignment to facilitate TAV-in-TAV and coronary re-access while maintaining low-profile access with a 14/16F expandable sheath.

The ongoing ALLIANCE study will assess the safety and efficacy of the new SAPIEN X4 THV in a broad patient population, including any surgical risk level, bicuspid anatomy, and valve-in-valve patients.^11^ The ALLIANCE prospective single-arm multicenter study in 915 patients includes a valve-in-valve (N=150) and bicuspid registry (N=150). VARC-3 outcomes at 30-days and 1-year will be compared to a historical control consisting of patients from the PARTNER 2 and PARTNER 3 trials.^11^

### Evolut FX

The latest iteration of the Evolut THV is the fourth-generation Evolut FX (EFX) SE (Medtronic) THV, which entered the market in 2022, providing a more symmetric and predictable final implant depth after release. The main features of this bioprosthesis, similar to its third-generation predecessor, the Evolut Pro+, are:

1. A nitinol delivery catheter capsule that allows recapturing and repositioning of the THV during deployment, and a nitinol design at the annulus that optimizes radial force.
2. A tall porcine pericardial sealing skirt with an external porcine pericardial wrap to reduce PVL.
3. An inline sheath, allowing TAVI in a wide range of vascular anatomies with a 14F delivery system for 23, 26, and 29 EFX implantation and 18F for 34-mm EFX implantation.

**Figure 2:**
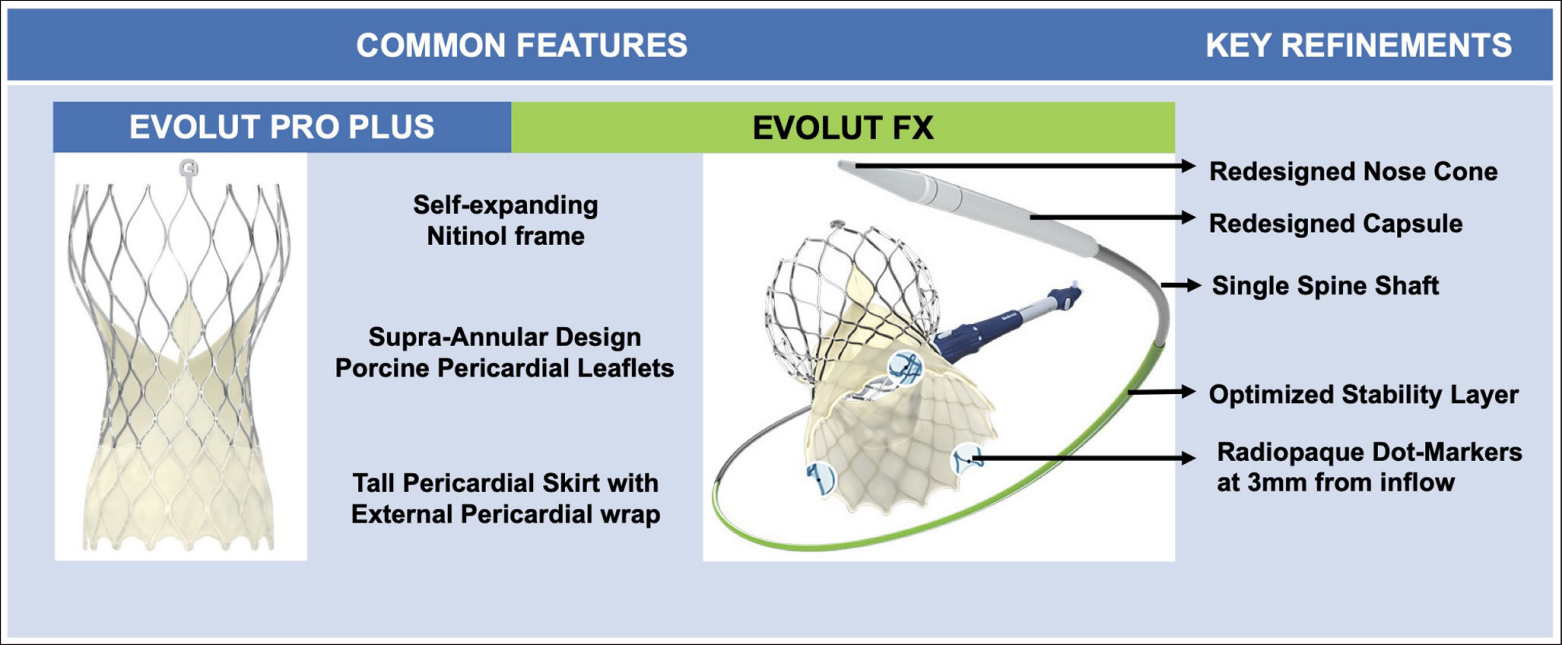
Novel features in the Evolut Pro Plus versus the Evolut FX transcatheter heart valves.

Despite an improved deployment technique and reduced permanent pacemaker implantation and PVL with the third-generation Evolut Pro+, achieving predictable implant depth and commissural alignment remained challenging. Innovations in the Evolut FX system were made to address these challenges, and it now has the following novel features compared to the PRO+ system (Figure 2)^12^,^13^,^14^,^15^,^16^:

1. Single spine (FX) versus 2 spines (PRO+) to improve delivery flexibility and trackability.
2. Improved nose cone design for smoother femoral artery insertion via Inline sheath, and atraumatic vascular access.
3. Enhanced visualization during deployment, with 3 radiopaque “Dot” markers at 3 mm from inflow to denote 3 commissural locations, thus improving commissural alignment and achieving a more symmetric deployment.
4. An optimized stability layer for more predictable valve deployment.

EFX is approved in four sizes: the 23-, 26-, and 29-mm can pass through arteries down to 5 mm, while the 34-mm system can pass down to 5.5 mm. The first-in-human single-center experience of the newest Evolut FX TAVR system showed good outcomes with a more symmetric implant depth and improved commissural alignment over the PRO+ system.^17^ In a multicenter experience with the Evolut FX system, significant advancements in THV and delivery system technology were observed within the Evolut platform over its predecessors, with improved commissural alignment, more symmetric device implant despite infrequent use of the Lundiquist stiff wire, reduced device recapture, and improved PVL while maintaining similar excellent hemodynamic performance.^18^ Future iterations of the Evolut platform aim to increase cell size and further optimize the ease of coronary and commissural alignment.

### Portico Navitor

**Figure 3:**
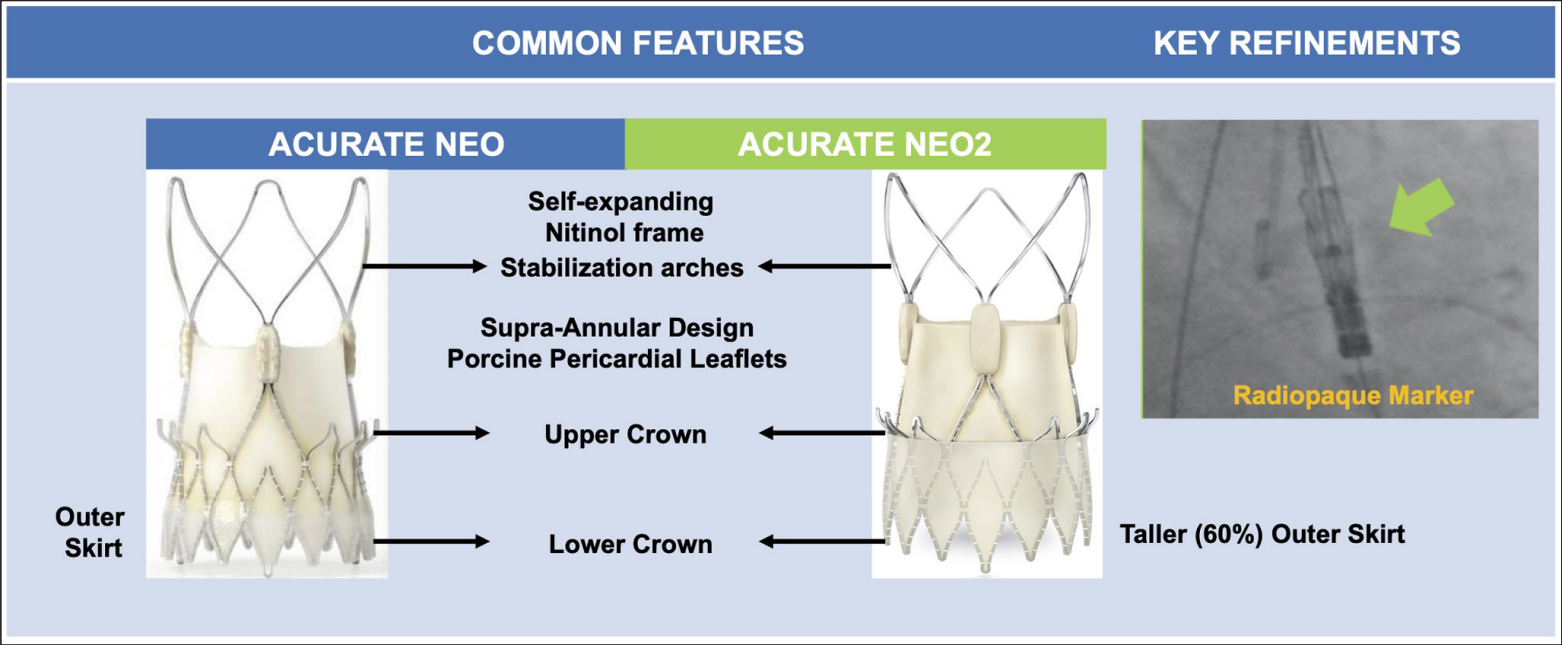
Novel features in the Portico versus the Portico Navitor transcatheter heart valves.

The third generation Navitor THV is the latest iteration and successor of the Portico valve (Abbott Structural Heart). Similar to Portico, the Navitor is a self-expanding, fully resheathable, and partially retrievable THV consisting of three bovine pericardial tissue valve leaflets mounted in a radiopaque nitinol frame with an intra-annular design to facilitate future coronary access. The Navitor has the following key refinements compared with Portico (Figure 3):

1. Large cell design to minimize coronary obstruction and improve coronary access, and curved aortic cells to reduce injury to native structures.
2. Dedicated inner and outer NaviSeal fabric cuffs, which actively synchronize to the cardiac cycle in order to fill the calcification-related gaps between the THV and virtual basal ring, and a landing zone without cutouts to improve sealing and reduce PVL.
3. Induction of a 14F low-profile FlexNav delivery system with improved deliverability and feasibility in patients with small peripheral access (up to 5 mm) and lower vascular complication rates.

The available valve sizes include 23 mm, 25 mm, 27 mm, and 29 mm, allowing treatment of aortic valves with an annulus perimeter ranging from 60 mm to 85 mm. In the recent prospective, multicenter, single-arm Portico NG study evaluating the safety and performance of the new Navitor THV in 120 high or extreme surgical risk patients, Navitor implantation was associated with excellent outcomes including procedural success in 97.5% and 30-day mortality or > mild PVL, with none/trace PVL in 80%.^19^ Additionally, the large effective orifice area (EOA) and low, single-digit transvalvular gradients are comparable to supra-annular THVs and lower compared to other intra-annular THVs. With encouraging results from the Portico NG approval study, the Navitor THV is now approved by the US Food and Drug Administration for high and extreme surgical-risk patients, and patients enrolled will be followed annually over 5 years. Outcomes in low-to-intermediate-risk patients treated by Navitor will be reported by the VANTAGE international pre-market clinical trial, which is currently enrolling in Europe and Australia, and is expected to start enrolment in the US in the first half of 2023.^20^

### Acurate NEO2 THV

The latest iteration of the neo THV and evolution of the ACURATE Neo is the ACURATE neo2 (NEO2, Boston Scientific), a self-expanding THV with porcine pericardial tissue bioprosthesis sewn onto a self-expanding nitinol stent. Similar to its predecessor, the valve has a supra-annular design conferring an excellent hemodynamic profile, and includes three stabilization arches for axial alignment and upper and lower anchoring crowns for accurate positioning during a top-to-bottom deployment. An open upper frame provides unrestricted coronary access. The NEO2 sizing as well as crimping, loading, and release mechanism (top-down) remain unchanged compared with the original Neo THV.

**Figure 4:**
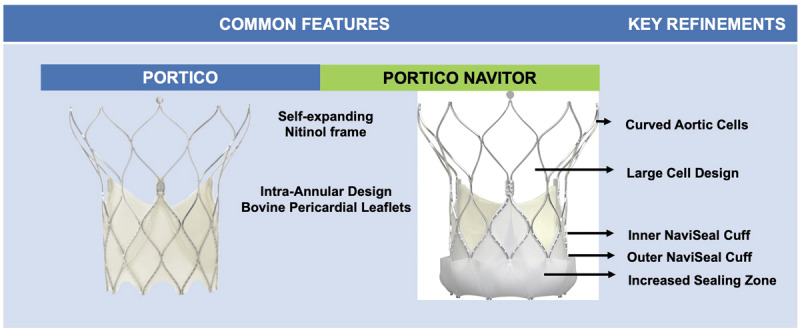
Novel features in the ACURATE neo versus the ACURATE neo2 transcatheter heart valves.

The main limitation of the initial ACCCURATE neo design was the high incidence of ≥ moderate PVL due to its relatively low radial force and the absence of a high sealing skirt.^21^,^22^ The next generation NEO2, which became commercially available in Europe in 2020, was redesigned with the following important modifications while preserving the aforementioned favorable device characteristics (Figure 4):

1. Approximately 60% taller pericardial sealing skirt, extending to the upper crown reaching the waist of the THV stent, for improved sealing and reducing PVL.
2. New radiopaque positioning marker to enhance visualization and accuracy during THV deployment.
3. Flexible NEO2 delivery catheter with a new, less traumatic tip design.
4. A low profile 14F expandable introducer sheath (iSleeve^TM^) that can accommodate all three valve sizes and a wide range of anatomies.
5. Easily identifiable commissural posts at the base of the stabilizing arches that facilitate near universal commissural alignment, which can be assessed in the right-left cusp overlap view.

The NEO2 is available in three different sizes to treat aortic valve annuli between 21 mm to 23 mm, 23 mm to 25 mm, and 25 mm to 27 mm, respectively. Promising data on real-world outcomes with the NEO2 THV was recently presented from two European registries, which showed excellent procedural success with low mortality rates in addition to excellent hemodynamics with a marked reduction in PVL and permanent pacemaker (PPM) rates compared with the first-generation neo device.^23^,^24^ The lower radial force of the nitinol THV frame leads to less mechanical trauma to the conduction system and may explain the lower device-related PPM. This, however, is at the expense of greater need for pre- and post-dilatation, particularly in cases with severe valve calcification. Other important device limitations include lack of repositionability when compared with other supra-annular THVs, and lack of coverage of the entire spectrum of annular sizes when compared with other contemporary THVs. The NEO2, however, is still under investigation in the US in the ongoing ACURATE IDE trial that will evaluate the NEO2 THV versus other THVs in all risk categories.^25^ It is currently 50 low-risk patients away from completion. Additionally, a larger valve covering annular sizes 27 mm to 30 mm, the Acurate XL, is currently enrolling in a registry arm of the ACURATE trial.

### TAVI for AR: JenaValve

TAVR in pure severe aortic regurgitation (AR) with nondedicated devices has been challenging due to valve embolization, residual postprocedural AR, and need for a second THV.^26^ Considering suboptimal outcomes when compared with TAVR for AS, the treatment of pure AR clearly calls for dedicated device technology that can provide reliable annular anchoring in the absence of aortic valve calcium. The JenaValve (JenaValve Technology) has emerged as a dedicated THV solution for treating the pure AR population, with its latest iteration, the JenaValve trilogy, having undergone several refinements since its first in-human implantation in 2011.^27^,^28^

**Figure 5:**
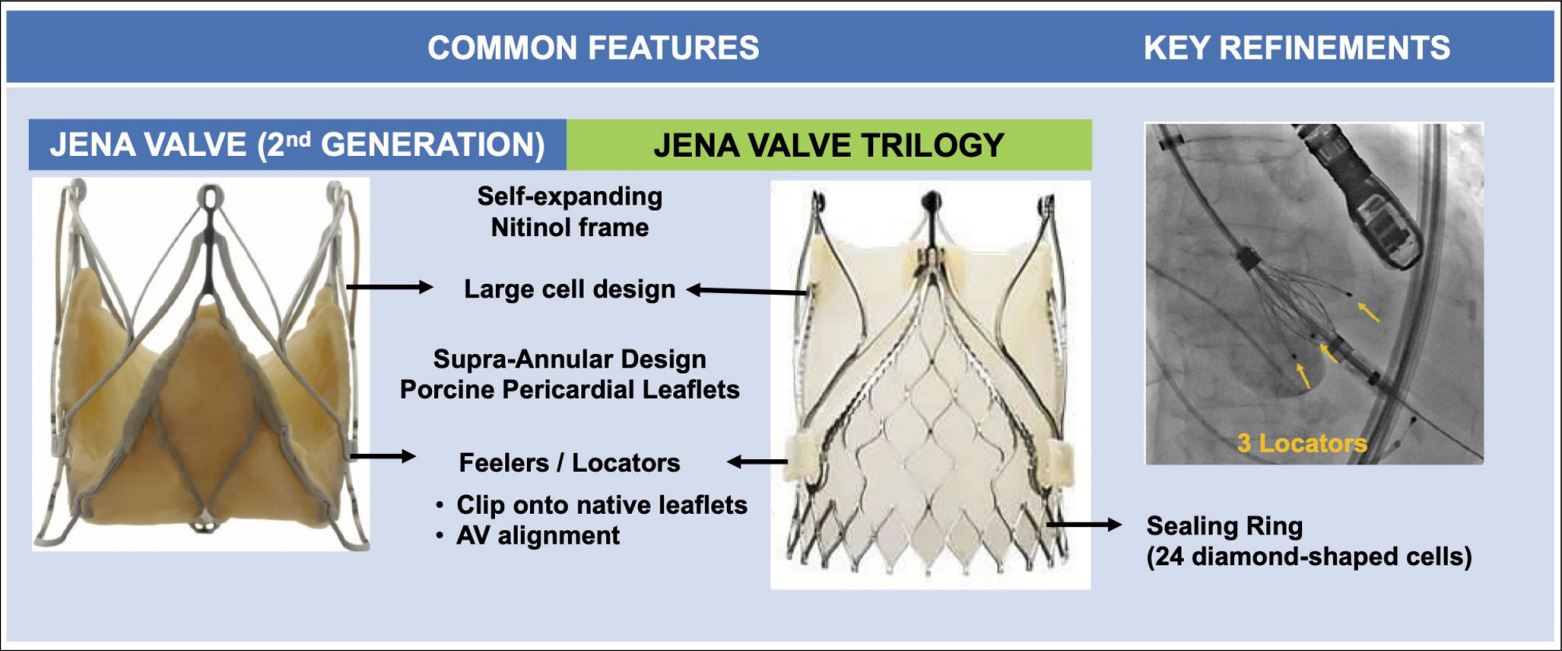
Novel features in the JenaValve second generation versus the JenaValve Trilogy.

The JenaValve is available in three sizes: 23 mm, 25 mm, and 27 mm, allowing treatment of aortic valves with an annuli ranging from 21 mm to 27 mm. THV oversizing of 10% to 20% to annular dimension is recommended for adequate sealing in pure AR. The device is delivered using an 18F equivalent Coronatix catheter positioned in the ascending aorta. The JenaValve trilogy has the following key features (Figure 5):

1. Porcine pericardial leaflets with a supra-annular design mounted on a self-expanding nitinol support frame.
2. Large cell design with 27F to 31F opening to minimize coronary obstruction and improve coronary access.
3. Three integrated radiopaque locators (previously referred to as “feelers”) that enhance visualization during deployment, allowing for a more precise implant depth; align with sinuses and improve commissural alignment; and also serve as a strut onto which the nitinol frame is expanded, essentially clipping the device to the native leaflets thereby firmly anchoring the THV independent of cusp calcification and mitigating risk of migration.
4. Sealing ring of 24 diamond-shaped cells that provide annular conformability and sealing, and THV securement with minimal protrusion into the LVOT.
5. Redesigned introducer system that has allowed for transfemoral implantation, with the transapical device no longer available.

The first in-human transfemoral JenaValve implantation for pure AR was successfully performed in 2017.^29^ The transfemoral system is currently under investigation in two trials, one for AR (ALIGN-AR)^30^ and the other for AS (ALIGN-AS).^31^ In the first prospective data from the ALIGN-AR trial—which evaluated transfemoral TAVI with JenaValve in 70 patients with pure AR—device success was achieved in 94.3%, with one patient having THV embolization and another requiring conversion to open cardiac surgery.^32^ While 30-day mortality (2.9%) and stroke-rates (2.9%) were comparable to severe AS patients undergoing TAVR, the pacemaker implantation rate was high at 23%.

## Structural Valve Degeneration and Novel Leaflet Technologies

The biological tissue used in bioprosthetic valves, including the existing THV, are prone to structural valve degeneration (SVD), resulting in limited long-term durability. SVD is a progressive process starting with morphologic changes causing leaflet deterioration that eventually culminates in hemodynamic deterioration with stenosis, regurgitation, or both. The development of early SVD is thought to be related to prosthesis characteristics, such as stent and leaflet design, patient-prosthesis mismatch, and transvalvular gradients; additional clinical risk factors may include young patient age, renal failure, subclinical valve thrombosis, or abnormalities of calcium metabolism or anticoagulation cascade.^33^,^34^ As TAVR expands to younger and lower-risk patients with longer life expectancy, the long-term THV durability is becoming increasingly important because their life expectancies will likely exceed the durability of the THV. To address the issue of bioprosthesis durability, newer bioprosthetic valve platforms are being developed and tested, focusing on innovations in leaflet material and design to improve durability profiles. Hence, we provide this update on the key advancements in leaflet technology.

### RESILIA Tissue

SVD in bovine pericardial valves typically manifests as leaflet calcification, resulting in stiffening and subsequent restenosis, with THV stenosis being the primary mode of SVD impacting hemodynamic performance in the PARTNER 2 VIV and VIVID Registries.^35^,^36^ While multiple factors influence tissue calcification, tissue exposure to free aldehydes during glutaraldehyde fixation and storage is a major source of calcification.^37^ RESILIA tissue (Edwards Lifesciences) is bovine pericardial tissue incorporating a novel integrity preservation anticalcification technology that includes stable capping of free aldehydes, preventing calcium binding and glycerolization while preserving and protecting leaflet tissue. It also enables dry storage while maintaining thickness and elasticity of bovine pericardial leaflets.

RESILIA tissue was initially incorporated in the Edwards’ surgical valve platforms, including the commonly implanted INSPIRIS RESILIA aortic valve, MITRIS RESILIA mitral valve, and KONECT RESILIA aortic valve conduit (Edwards Lifesciences). The safety and hemodynamic performance of the INSPIRIS bioprosthetic aortic valve with RESILIA tissue showed promising results through 5 years in the COMMENCE trial, with clinically stable hemodynamics, minimal regurgitation, and no evidence of SVD.^38^ Given the inevitability of THV reintervention with the expansion of TAVR in younger patients with longer life expectancies, the potential implications of a more durable tissue valve are far-reaching. The RESILIA valve has now been incorporated into the Edwards THV platforms, with the SAPIEN 3 Ultra RESILIA valve made available in the US in limited release in the fourth quarter of 2022. The RESILIA tissue will be incorporated in the next generation of the SAPIEN X4 THV prosthesis and may further tip the scales towards a more durable THV option.

### Anteris DurAVR Single-Piece 3D Leaflet TAVR Technology

The DurAVR THV System (Anteris Technologies) is a novel balloon-expandable THV platform specifically designed to address the issue of hemodynamic performance, THV durability, and AV reintervention. In contrast to the traditional existing THV platforms that consist of three pieces of leaflet tissue sewn onto a frame, the novel feature of this platform’s leaflet technology is its unique three-dimensional single-piece leaflet design molded to mimic native AV anatomy. Computational fluid modeling comparing the two leaflet designs, has shown the single-piece leaflet design to have a greater coaptation area with improved coaptation height, with a more uniform distribution of leaflet stress resulting in optimal laminar flow and lesser leaflet strain.^39^ The leaflet design also allows for a shorter stent height with a large open cell geometry while maintaining a large EOA. In addition, temporal risk of leaflet calcification and SVD is further mitigated by advancements in leaflet tissue formulation by using DNA- and glutaraldehyde-free acellular bovine pericardial leaflet tissue that is processed with the ADAPT tissue engineered anticalcification technology.^40^,^41^

In the first-in-human, prospective, nonrandomized, single-arm, single-center study designed to evaluate the safety and feasibility of the DurAVR THV in 13 patients, procedural success was 100% with no mortality, disabling stroke, or life-threatening bleeding and excellent native-like 30-day hemodynamic performance in the first five patients who completed follow-up.^42^ With potential implications on hemodynamic performance and THV durability, the DurAVR THV leaflet technology may represent the next-generation of THV as TAVR expands to treat younger, lower-risk patients earlier in the disease process. The DurAVR US early feasibility study to assess the acute and long-term (10-year) safety and feasibility of the DurAVR device will begin enrolling in the second quarter of 2023.^43^

### Foldax Tria Heart Valve

To overcome the issue of SVD with bioprosthetic valves, various polymers have been evaluated as candidates for valve leaflet material to engineer the “ideal” heart valve that will potentially last a patient’s lifetime. In contrast to the biological pericardial leaflet tissue in the bioprosthetic valves, polymer properties can be controlled and the geometry of polymer valve leaflets can be designed to ensure physiological function, using formulations that are biocompatible, biostable, and have high biomechanical strength. A biomedical-grade siloxane-based polyurethane-urea (TRIA LifePolymer) has recently been developed and undergone extensive in vitro and in vivo testing as polymer-based valve leaflet material.^44^ The TRIA LP heart valve comprises three flexible LP leaflets cast onto a radiovisible polyether-ether ketone stent with a polytetrafluoroethylene felt sewing ring. The automated robotic manufacturing process can achieve an exceptional level of precision, ensuring that critical measurements such as leaflet thickness are within micrometer tolerances. Additionally, the valve can be stored in a dry state and does not require any preparation before implantation.

The first in-human early feasibility study in 15 patients undergoing SAVR demonstrated marked and sustained improvements in transvalvular gradients, valve EOA, and New York Heart Association functional class to 1 year following valve implantation.^45^ With encouraging results at 3-year follow-up in > 50 implants to date, large-scale clinical trial evaluation of the TRIA LP heart valve is currently underway and the technology is being incorporated in the THV platform. The Foldax early feasibility study, with plans to enroll up to 40 patients and follow up for 5 years after implantation, is expected to start enrollment in 2023.^46^

## Future Directions

Despite advancements in device technology and leaflet design, several issues remain to be solved as TAVR indications continue to expand. While PVL appears to be decreasing with improvement in THV design, newer-generation prostheses will have to address the issue of pacemaker implantation that is still substantial at 10.8%.^4^ Stroke incidence has remained low at 2.3% but is still concerning considering its adverse impact. Additional data on the effectiveness of cerebral embolic protection devices during TAVR are forthcoming from ongoing randomized trials, in particular the BHF PROTECT-TAVI (British Heart Foundation Randomized Trial of Routine Cerebral Embolic Protection in Transcatheter Aortic Valve Implantation).^47^ The role of TAVR in patients with concomitant coronary artery disease also needs to be better defined.

## Conclusions

Advances in TAVR have transformed the treatment landscape of patients with AS over the past two decades owing to a constant evolution in procedural techniques and device technology. While the next generation of THV devices are proving to be safer and more effective than their predecessors, a greater focus on the lifetime management of aortic stenosis is directed at the latest device iterations as younger and lower-risk patients are likely to outlive their THV.

## Key Points

- Expansion of transcatheter aortic valve replacement across the entire spectrum of surgical risk has seen a continuous evolution in device technology.
- The latest iterations of the various transcatheter heart valve (THV) platforms are proving to be safer and more effective than their predecessors.
- As younger and lower risk patients are likely to outlive their THV, a greater focus is now placed on the lifetime management of aortic stenosis.
- Ongoing innovations are evolving in leaflet material and design to improve THV durability profiles and engineer the “ideal” heart valve to potentially last a patient’s lifetime.
